# Primary lymphocutaneous nocardiosis in an immunocompetent patient

**DOI:** 10.1186/1476-0711-3-24

**Published:** 2004-11-15

**Authors:** Sofia Maraki, Stavros Chochlidakis, Eleni Nioti, Yannis Tselentis

**Affiliations:** 1 Department of Clinical Bacteriology, Parasitology, Zoonoses and Geographical Medicine, University Hospital of Crete, 712 01 Heraklion, Crete, Greece; 2 Department of Orthopedics, University Hospital of Crete, 712 01 Heraklion, Crete, Greece

## Abstract

**Background:**

*Nocardia brasiliensis *is a rare human pathogen usually associated with localized cutaneous infections.

**Case Presentation:**

We report a case of primary lymphocutaneous *Nocardia brasiliensis *infection developed after a bone fracture of the left hand of an otherwise healthy 32-year-old man. Treatment with trimethoprim-sulfamethoxazole given for a total of three months combined with surgical debridement resulted in complete resolution of the infection.

**Conclusion:**

Nocardiosis should be part of the differential diagnosis in patients with sporotrichoid infection, particularly those with a history of outdoor injury. Culture of the affected tissue and antimicrobial susceptibility testing of the isolate should be performed for diagnosis and treatment.

## Background

Nocardiosis is a rare localized or systemic infection caused by several species of the genus *Nocardia*. This genus consists of strictly aerobic, Gram-positive, variably acid-fast, filamentous bacteria with a tendency to fragment into bacillary and coccoid forms [1]. *N. asteroides*, *N. farcinica*, *N. nova *(included in the *N. asteroides *complex) and *N. brasiliensis *are the species most often involved in human disease [1,2]. *N. brasiliensis *has been recovered from the soil in many tropical and subtropical areas but rarely in temperate areas. Traumatic inoculation of *N. brasiliensis *into the skin is the most typical mode of acquisition of the infection due to this organism [1,2].

Herein, we describe a case of a man, who had an accident at work and 1 week later developed lymphocutaneous infection caused by *N. brasiliensis*.

## Case Presentation

A previously healthy 32-year-old man was referred to the emergency department of orthopedics with traumatic injuries of the index, middle and ring fingers of the left hand. The injury happened at work while he was operating a machine of cotton elaboration.

On admission, routine laboratory investigations showed only elevated white blood cell count (12,400/mm^3^), while red blood cell count, haemoglobin, chemistry and urine analysis were within normal limits. Radiography of the bones of his left hand revealed fractures of the nail bones of the middle and the ring finger. Surgical debridement of the damaged soft tissue was undertaken with amputation of these two nail bones. The patient was hospitalized and intravenous therapy with ceforanide (1 g/12 h), ofloxacin (200 mg/12 h) and metronidazole (500 mg/8 h) was initiated.

Four days after his admission the injury of the hand became tender, erythematous, swelling and began to drain. The purulent material expressed from the hand was sent for culture. Three days later the lesion worsened and was complicated with lymphangitis. The patient was noted to be febrile (38.5°C) without any other systemic symptoms.

Physical examination revealed multiple erythematous subcutaneous nodules along the lymphatics extending up the patient's left forearm. These nodules were tender and painful. There was no regional lymphadenopathy. Debridement of the lesions was performed and the tissue was submitted for bacterial and fungal cultures. The Gram-stained smear showed polymorphonuclear leucocytes and Gram-positive fine, branching filaments, partially acid-fast, with a tendency to fragment into coccoid and bacillary forms.

Laboratory tests showed: white blood cell count (18,000/mm^3^), absolute neutrophil count, 12,780/mm^3^, erythrocyte sedimentation rate, 75 mm/h; and C-reactive protein, 14.2 mg/dl (normal < 0.8 mg/dl).

**Figure 1:**
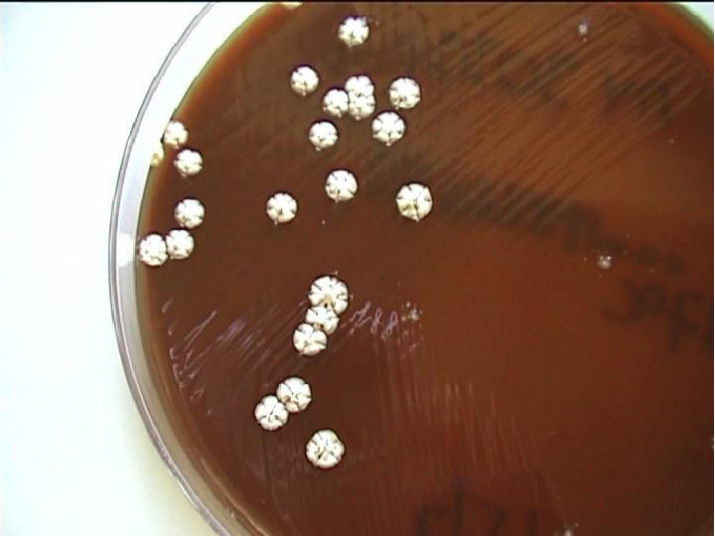
Rough chalky-white colonies of *Nocardia brasiliensis *grown on Columbia blood agar

After 5 days of incubation cultures of the pus and the tissue on Columbia blood agar grew white colonies adherent to the agar, rough with a velvety surface, having a characteristic mouldy odor (Figure 1). Colonial characteristics, physiological properties and biochemical tests performed identified the isolate as *Nocardia brasiliensis *(Table 1). Susceptibility to the antibiotics by the determination of the MICs using the E-test method (AB Biodisk, Solna, Sweden), showed that the isolate was sensitive to trimethoprim-sulfamethoxazole, amoxicillin-clavulanic acid, gentamicin, tobramycin, amikacin, and minocycline, intermediate to ciprofloxacin and resistant to ampicillin, second and third generation cephalosporins, erythromycin, clindamycin, ofloxacin and pefloxacin. The patient's antimicrobial therapy was changed to intravenous trimethoprim-sulfamethoxazole (160/800 mg b.i.d). The patient responded to therapy. Following 2 weeks of treatment the patient improved and all laboratory tests returned to normal. He was discharged 3 weeks after his admission on oral trimethoprim-sulfamethoxazole (160/800 mg b.i.d). The antibiotic therapy was continued for a total of 3 months. His hand and arm lesions were healing well and 6 months later revealed complete resolution of the infection without signs of recurrence.

**Table 1:** Physiological characteristics and biochemical reactions of our *Nocardia brasiliensis *isolate

**Test or characteristic**	**Reaction of our strain**
Decomposition of:	
Adenine	-
Casein	+
Tyrosine	+
Xanthine	-
API 20C AUX assimilation results:	
Glucose	+
Glycerol	+
Galactose	+
N-acetyl-D-glucosamine	+
Inositol	+
Adonitol	-
Trehalose	+
Equivalent growth at 35°/45°C	-
Lysozyme broth	+
Production of arylsulfatase (7d)	-
Gelatin liquefaction (7d)	+
Sensitivity to:	
Gentamicin	+
Tobramycin	+
Amikacin	+
Erythromycin	-

## Discussion

In the United States, there are an estimated 500–1,000 new cases of nocardiosis each year [3]. On the basis of epidemiological surveys conducted in France and Italy, the annual estimated incidence of human nocardiosis is 150–250 and 90–130 cases, respectively [4,5]. *N. brasiliensis *accounts for only about 7–14% of the reported cases [3]. The incidence of nocardiosis in Greece is still unknown because the number of nocardial infections are not reported to the public health authorities. Only one case of *N. brasiliensis *lymphocutaneous syndrome has been previously described in the same geographic area [6].

*N. brasiliensis *although rarely implicated in pulmonary and disseminated infections in immunocompromised patients, has been most commonly associated with cutaneous infections [7]. *Nocardia *enters the skin after traumatic inoculation injuries, varying from contaminated abrasions and puncture wounds to insect and animal bites. The most common resulting skin lesions are on the upper and lower extremities [7]. Cutaneous manifestations include: (i) mycetoma, (ii) lymphocutaneous (sporotrichoid) infection, (iii) superficial skin infection, and (iv) disseminated infection with cutaneous involvement.

The present case is consistent with the classical presentation of lymphocutaneous infection with a primary lesion at the site of injury on the hand and an ascending lymphangitis involving the forearm. The inoculation probably occurred from the cotton that had been contaminated by *Nocardia *and entered the wound after the accident.

The lymphocutaneous syndrome can be caused by a wide variety of microorganisms. The most common causative agents of the syndrome in addition to *Sporothrix schenkii *and *Nocardia brasiliensis*, include *Mycobacterium marinum *and *Leishmania *species. Less common causes are *Coccidioides immitis*, *Cryptococcus neoformans*, *Histoplasma capsulatum*, *Blastomyces dermatitidis*, *Pseudoallescheria boydii*, other species of *Mycobacterium*, *Streptococcus pyogenes*, *Staphylococcus aureus *and viruses as cowpox virus and herpes simplex [8]. A history of a traumatic wound contaminated with soil and the relatively brief incubation period (less than 2 weeks) suggest nocardiosis. Diagnosis of nocardial infection can be established by cultural isolation of the microorganism. Identification to the species level can be successfully performed either by conventional biochemical methods or by molecular techniques [1,9].

Trimethoprim-sulfamethoxazole combination is recognized the drug of choice for nocardiosis [10]. Primary lymphocutaneous nocardiosis may be curable after a course of 2 to 4 months, although several studies report clinical cures of cutaneous nocardiosis caused by *N. brasiliensis *after only 2 to 3 weeks of therapy. In patients with sulfa intolerance or those who fail therapy with trimethoprim-sulfamethoxazole, alternative therapy must be based on sensitivity testing. Minocycline, tetracycline, amikacin and amoxicillin-clavulanic acid have been successfully used [11].

Although rare, lymphocutaneous nocardiosis must be considered, diagnosed with appropriate cultures and adequately treated, in order to prevent progression to dissemination of the primary skin disease.
